# Characterizing Defects Inside Hexagonal Boron Nitride Using Random Telegraph Signals in van der Waals 2D Transistors

**DOI:** 10.1021/acsnano.4c06929

**Published:** 2024-09-28

**Authors:** Zhujun Huang, Ryong-Gyu Lee, Edoardo Cuniberto, Jiyoon Song, Jeongwon Lee, Abdullah Alharbi, Kim Kisslinger, Takashi Taniguchi, Kenji Watanabe, Yong-Hoon Kim, Davood Shahrjerdi

**Affiliations:** † Electrical and Computer Engineering, New York University, Brooklyn, New York 11201, United States; ‡ School of Electrical Engineering, Korea Advanced Institute of Science and Technology (KAIST), 291 Daehak-ro, Daejeon, Yuseong-gu 34141, Korea; § Microelectronics and Semiconductor Institute, King Abdulaziz City for Science and Technology (KACST), Riyadh 11442, Saudi Arabia; ∥ Center for Functional Nanomaterials, Brookhaven National Laboratory, Upton, New York 11973, United States; ⊥ Research Center for Materials Nanoarchitectonics, National Institute for Materials Science, 1-1 Namiki, Tsukuba 305-0044, Japan; # Research Center for Electronic and Optical Materials, National Institute for Materials Science, 1-1 Namiki, Tsukuba 305-0044, Japan

**Keywords:** defect engineering, hBN dielectric, random telegraph signal, low-frequency noise, low-disorder heterostructure, nonradiative multiphonon model

## Abstract

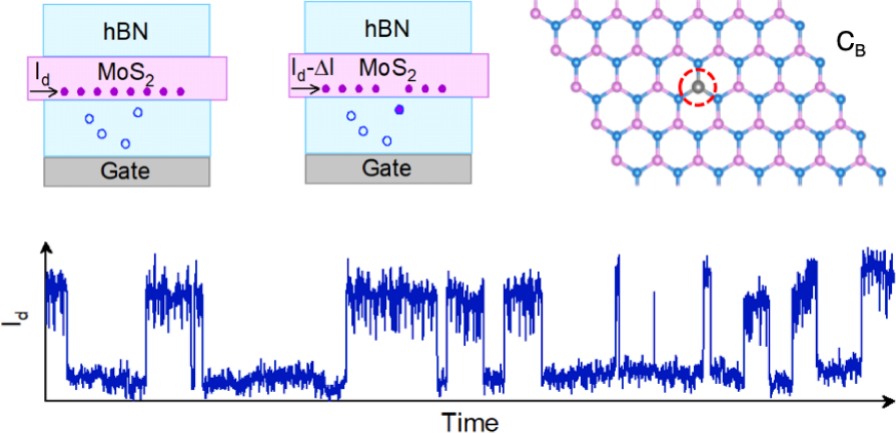

Single-crystal hexagonal boron nitride (hBN) is used extensively in many two-dimensional electronic and quantum devices, where defects significantly impact performance. Therefore, characterizing and engineering hBN defects are crucial for advancing these technologies. Here, we examine the capture and emission dynamics of defects in hBN by utilizing low-frequency noise (LFN) spectroscopy in hBN-encapsulated and graphene-contacted MoS_2_ field-effect transistors (FETs). The low disorder of this heterostructure allows the detection of random telegraph signals (RTS) in large device dimensions of 100 μm^2^ at cryogenic temperatures. Analysis of gate bias- and temperature-dependent LFN data indicates that RTS originates from a single trap species within hBN. By performing multispace density functional theory (MS-DFT) calculations on a gated defective hBN/MoS_2_ heterostructure model, we assign substitutional carbon atoms in boron sites as the atomistic origin of RTS. This study demonstrates the utility of LFN spectroscopy combined with MS-DFT analysis on a low-disorder all-vdW FET as a powerful means for characterizing the atomistic defects in single-crystal hBN.

## Introduction

Single-crystal hexagonal boron nitride (hBN) plays a significant role in many research studies involving two-dimensional (2D) materials. A notable application of the insulating hBN is in constructing van der Waals (vdW) heterostructures by encapsulating 2D materials between hBN flakes.^1^ In these heterostructures, the high purity and excellent crystal quality of hBN have made it an *ideal* insulator for 2D materials, leading to significant enhancements in carrier transport properties.^2^ However, native disorders in hBN layers, despite their low density, have been identified as a limiting factor for further enhancing carrier transport in graphene heterostructures^3^ and the performance of 2D transistors.^4,5^ On the other hand, harnessing defects in hBN has proven beneficial for implementing device concepts such as random number generators,^6,7^ and single-photon emitters (SPE).^8−12^ Therefore, understanding the nature of defects in hBN, including dynamics of their charging and discharging, is of great importance for advancing 2D electronics and quantum technologies.

Early material characterizations of hBN crystals using cathodoluminescence and elemental analysis revealed the presence of vacancies and impurities such as carbon and oxygen.^13−15^ The incorporation of carbon and oxygen in hBN was also revealed through direct imaging using electron microscopy on monolayer hBN exfoliated from bulk samples.^16^ Further insights into the nature of individual native defects within hBN were obtained through direct visualization using scanning tunneling microscopy (STM), which revealed three distinct charged defect states within its bandgap.^17^ This finding aligns with a recent study by Hayee et al.,^9^ which links the distinct emission spectra observed in hBN SPE with four defect candidates. In the effort to clarify the identity of localized native defects in hBN, first-principles calculations have been instrumental by providing information on their formation energies and associated defect energy levels.^18−22^ However, uncertainty persists regarding the identity and behaviors of defects in hBN due to the wide variety of possible defects and the absence of direct experimental evidence.

Characterizing the dynamics of charging and discharging of defects in an electronic device configuration is a powerful approach for understanding their nature. A commonly used method involves examining the bias and temperature dependence of random telegraph signal (RTS) noise in field-effect transistors (FETs),^23−26^ providing detailed insight into the energy and spatial distribution of defects. This approach has been extended to study 2D FETs with conventionally disordered dielectrics, such as SiO_2_ and Al_2_O_3_.^27−29^ Due to the abundance and variety of defects in conventional dielectrics, detecting RTS typically requires the use of downsized FETs (submicron dimensions) to minimize the number of defects in the channel area. In contrast, all-vdW 2D transistors obtained from hBN encapsulation offer a low-disorder electronic system.^30−33^ While their utility in transport studies have been demonstrated, their potential for the analysis of defects in hBN remains unexplored, which is the primary objective of this study.

In this study, we utilize hBN-encapsulated and graphene-contacted all-vdW MoS_2_ FETs as the experimental platform for low-frequency noise (LFN) studies. The low disorder of the electronic system in this fabricated device allows for the observation of random discrete level switching of current in large device dimensions of 100 μm^2^ at cryogenic temperatures. Analysis of the gate bias- and temperature-dependence of data indicates that RTS originates from a single energy state spatially located inside hBN near the MoS_2_/hBN interface. With the aid of multispace constrained-search density function theory (MS-DFT) calculations,^34,35^ we assign the carbon atom substituted into the boron site (C_B_) as the possible defect origin of experimental RTS observations.

## Results

### hBN-Encapsulated MoS_2_ FETs as a Test Platform

The STM analysis by Wong et al. revealed three energetically distinct charged defect states in single-crystal hBN.^17^ Their study identified two negatively charged (acceptor) defect states and one positively charged (donor) defect state. Furthermore, this study demonstrated direct manipulation of these defect charge states with the STM tip, indicating that the acceptor states are closer to the valence band than the donor states are to the conduction band. Inspired by the electric field-controlled charging/discharging of these defect states, we designed experiments to utilize LFN spectroscopy in n-type FET devices with hBN gate dielectrics for probing the donor defect state of hBN. This experimental design is rooted in the ability of a FET to shift the defect energy level in hBN relative to the Fermi energy level E_F_ using a gate bias. Should this hBN-encapsulated FET device provide a sufficiently low-disorder electronic system, we expect that this experimental design allows the observation of RTS that encodes the identity of the defect states. Hence, a key requirement of this experimental design is to implement FETs with pristine vdW interfaces to fulfill this condition.

**Figure 1:**
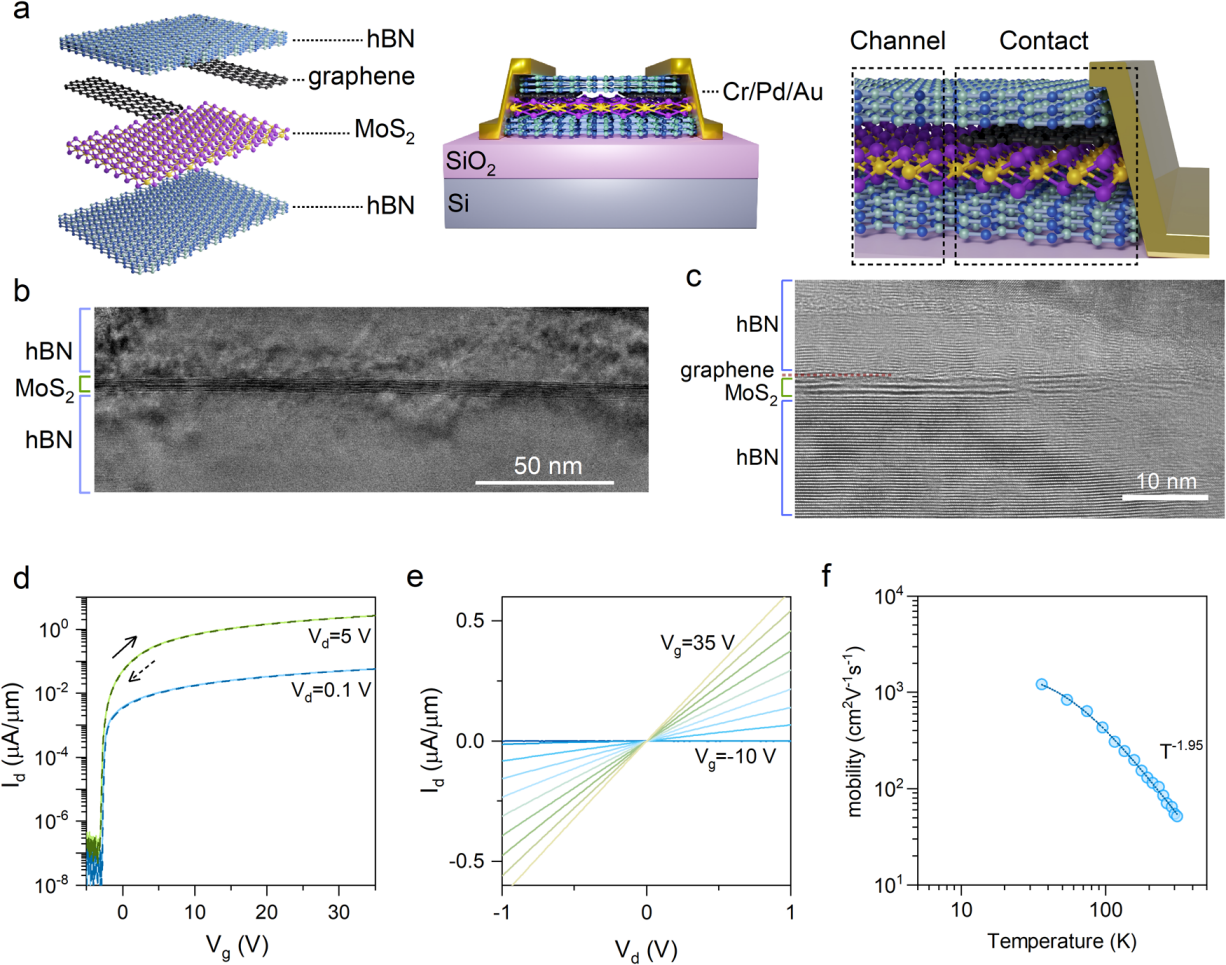
All-vdW MoS_2_ FET. (a) Schematic illustration of the hBN-encapsulated graphene-contacted MoS_2_ FET. The left panel schematically shows the individual constituents of the vdW heterostructure. The middle panel shows the final vdW FET. The magnified illustration in the right panel shows the contact and channel regions of the vdW MoS_2_ device. (b) Cross-sectional TEM image at the channel region, illustrating the hBN-encapsulated MoS_2_ heterostructure. (c) Cross-sectional TEM image at the edge contact region, illustrating the encapsulating hBN flakes, monolayer graphene contact, and few-layer MoS_2_. (d) Transfer characteristics and (e) output characteristics of the all-vdW MoS_2_ FET. The output characteristics are measured with a V_g_ increment of 5 V. (f) Four-point mobility as a function of temperature obtained by standard lock-in measurements.

Figure 1a shows the schematic of the vdW MoS_2_ FET experimental platform in a global back-gate configuration. Few-layer MoS_2_ was chosen as the channel material due to its n-type device character. This device architecture was adapted from a prior study, which demonstrates the effectiveness of vdW electrodes based on graphene in achieving low-resistance contacts to MoS_2_.^30^ With an optimal layer assembly process, the encapsulation in hBN can generate pristine vdW interfaces due to the atomically flat surface of the constituent 2D materials, while mitigating the carrier scattering from the surrounding environment.

While adapting this device architecture, our vdW stack fabrication process builds on our previously reported studies.^36,37^ We constructed the entire stack structure without the bottom hBN flake on an elastomeric stamp. Subsequently, the heterostructure fabrication was completed by high-temperature lamination of the stamp-supported stack onto the bottom hBN substrate to remove blisters due to interlayer interfacial contaminants.^37^ Using standard nanofabrication processes, the vdW stack was shaped to define the active region of the device, and the metal electrodes were formed through the edge contact with graphene,^1,38,39^ Details of the stacking and fabrication processes are described in Supplementary Note 1.

High-resolution transmission electron microscopy (HRTEM) was employed to investigate the interlayer interfaces within the MoS_2_ heterostructure, confirming atomically sharp interfaces. In Figure 1b, we show the HRTEM image of the FET channel region, consisting of the hBN-encapsulated MoS_2_. Figure 1c shows the edge contact region of the FET, where graphene overlaps with MoS_2_. The interface quality in the hBN-encapsulated MoS_2_ channel area at a larger scale was also inspected using Raman spectroscopy. These results indicate the spatial homogeneity and cleanliness of interfaces within the vdW heterostructure (see Supplementary Note 2 for Raman and electron dispersive X-ray spectroscopy results corresponding to the HRTEM).

Having established the excellent structural properties of the vdW heterostructure, we examined their electronic characteristics in a FET configuration. Figure 1d plots the drain current (I_d_) against the gate bias (V_g_) for a representative vdW MoS_2_ FET with channel width W = 6.6 μm and length L = 23 μm. The room-temperature transfer characteristics exhibit negligible hysteresis between the forward and backward sweeps, indicating low density of trap charges within the vdW heterostructure (see Methods for measurement conditions).

The room-temperature transfer characteristics also indicate a subthreshold swing (SS) of 100 mV/dec. Assuming a uniform distribution of charge traps within the bandgap, this SS value gives an estimated interface trap density D_it_ of 4 × 10^10^ eV^–1^cm^–2^ (See Supplementary Note 3 for details). This estimated D_it_ is 2 orders of magnitude lower than the optimal values in previously reported MoS_2_ FETs with disordered dielectrics, such as SiO_2_ and Al_2_O_3_.^27,28^ This analysis provides additional evidence about the excellent hBN-MoS_2_ interface within our MoS_2_ heterostructures. Furthermore, the plot in Figure 1e shows the output characteristic at low drain bias V_d_. The linear characteristics of the I_d_-V_d_ curves indicate a low-resistance ohmic contact provided by the graphene vdW electrodes.

The presence of charged impurities can severely limit carrier transport in 2D channel materials. Therefore, we used the carrier mobility as another proxy for evaluating the quality of the MoS_2_ heterostructure. The 4-point carrier mobility (μ_4 pt_) was extracted using standard lock-in measurements at high carrier densities (*n* > 4 × 10^12^ cm^–2^). At room temperature, the vdW MoS_2_ FET exhibits μ_4 pt_ = 55 cm^2^V^–1^s^–1^. To further examine the scattering mechanism of carriers in our devices, the variation of μ_4 pt_ with temperature is shown in Figure 1f. We fitted the measured μ_4 pt_ using the Matthiessen rule with two mobility components, μ_imp_ and μ_ph_, representing the effects of impurity scattering and phonon scattering, respectively. This analysis indicates that the carrier mobility at the high-temperature regime (>100 K) is limited mainly by the phonon scattering, following a power law dependence *μ*_*ph*_ ∼ T^–γ^ with γ = 1.95, consistent with the theoretical predictions for monolayer^40^ and bulk MoS_2_.^41^ At low temperatures (<100 K), however, the carrier transport is limited by impurity scattering. This analysis provides μ_imp_ ∼ 1800 cm^2^V^–1^s^–1^, which is consistent with state-of-the-art low-disorder vdW MoS_2_ FETs.^30^ The excellent carrier transport properties further support the high quality of the vdW MoS_2_ heterostructures in our experiments.

### Observation of RTS in vdW MoS_2_ FETs

The above material and electrical characterizations indicate that our vdW MoS_2_ FETs adequately fulfill the underlying condition for a low-disorder electronic system. Therefore, we next performed the LFN spectroscopy at different temperatures and gate biasing conditions.

**Figure 2:**
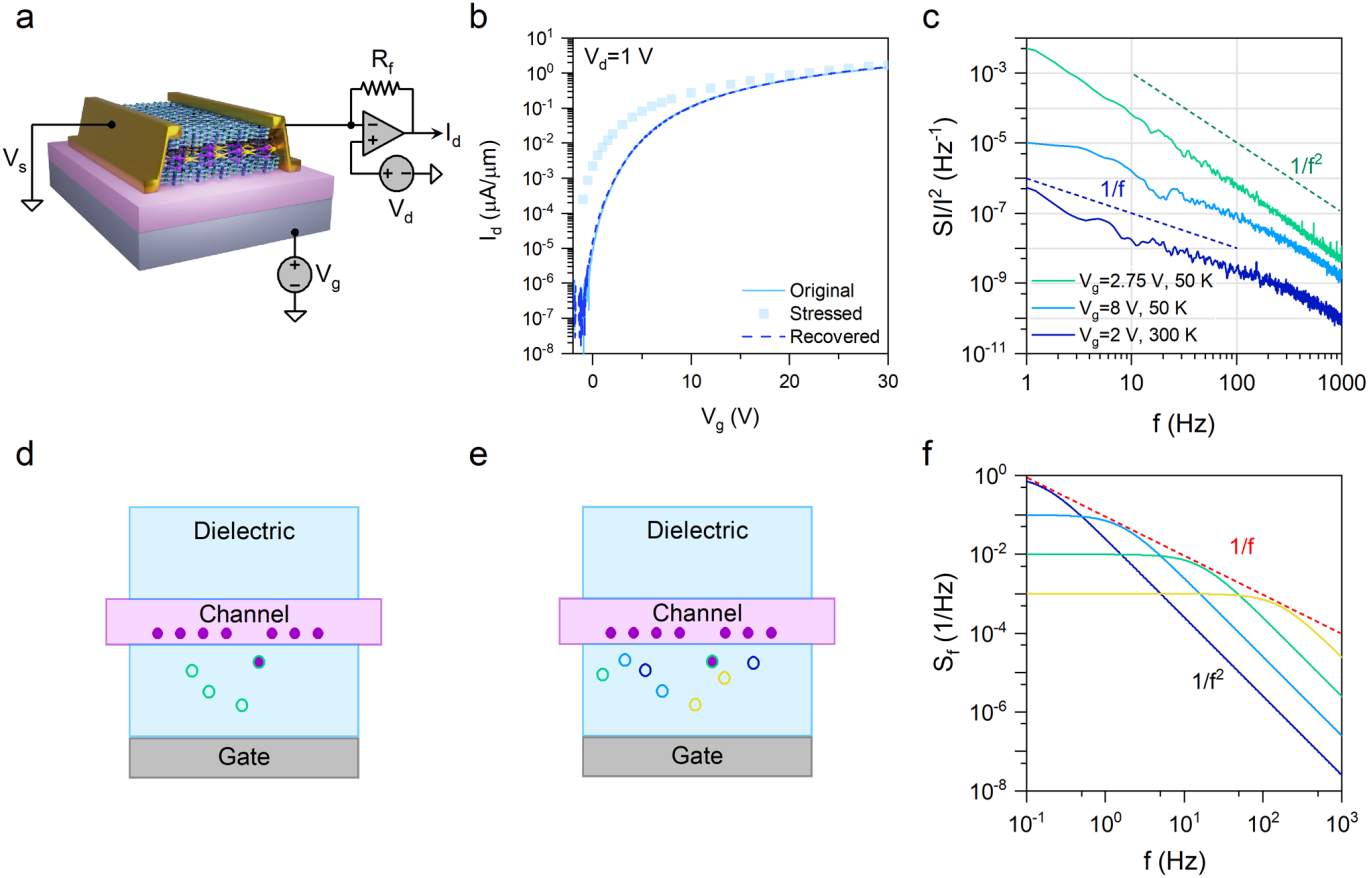
Low-frequency noise spectroscopy. (a) Schematic of the noise measurement setup. (b) I_d_-V_g_ characteristics illustrate negative V_th_ shift with V_g_ stressing and recovery after removing the V_g_ stressing. (c) Examples of PSD at different V_g_ and temperatures. At subthreshold V_g_ and low temperature, the PSD follows *1/f*^*2*^ trend (the green curve). At room temperatures or beyond the subthreshold regime, the PSD follows a *1/f* trend (the dark and light blue curves). Schematic illustration of electron trapping in a dielectric consisting of (d) a single defect species, and (e) multiple defect species. (f) The *1/f* spectrum (the red dashed line) can be represented as a summation of multiple *1/f*^*2*^ spectra (solid lines) with different corner frequencies. For illustration purposes, each *1/f*^*2*^ spectrum is color-coded to represent the effect of each distinct defect species in panel (e).

Figure 2a illustrates the schematic of the LFN measurement setup. Prior to LFN measurements, we initialized the device under test by depleting the defect sites within the dielectric (See Methods section). Each LFN measurement was performed by recording the drain current I_d_ for several minutes under a fixed gate V_g_ and drain V_d_ bias. These measurements were repeated at different temperatures for a series of different V_g_ and an identical V_d_ = 1 V.

Our initial observation from the LFN measurements was the negative shift of V_th_ (ΔV_th_) under positive gate bias stressing. To illustrate this, we reconstructed the I_d_-V_g_ characteristic of the device by plotting I_d_ from each sustained test against its corresponding V_g_ bias (square symbols). Figure 2b shows the representative data at 235 K, comparing this reconstructed I_d_-V_g_ curve against the transfer curve of the typical DC sweep (solid blue). The observed negative shift of V_th_ in this vdW FET under positive gate bias suggests an anomalous bias-temperature instability. We do not yet understand the origin of this observation, which merits further investigation in future studies. However, to decouple this effect from the LFN studies, presented next, all RTS data were obtained after the drain current of the device reached steady state under the fixed positive gate bias.

**Figure 3:**
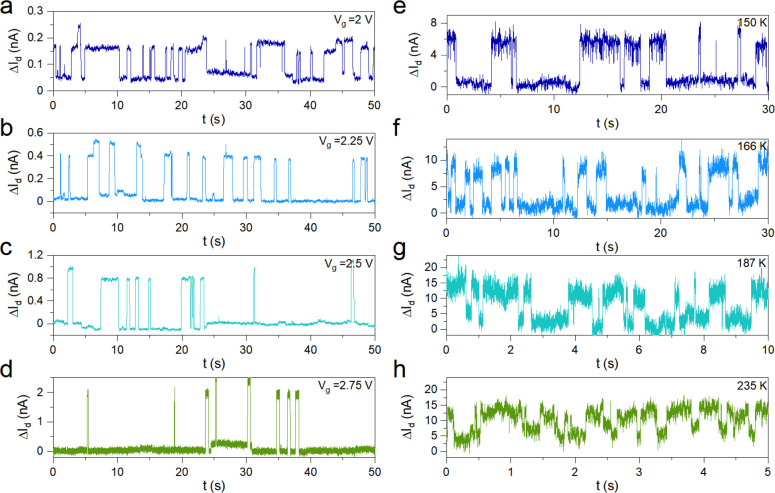
Random telegraph signal in all-vdW FET. (a)-(d) Discrete-level switching in I_d_ measured at a limited range of V_g_ close to the flat band at 50 K. (e)-(h) Discrete level switching in I_d_ measured at fixed V_g_ but different temperatures. Here, we plotted the difference between the discrete levels (ΔI_d_).

To analyze the LFN data, we initially examined the frequency dependence of the LFN characteristics by applying the fast Fourier transform (FFT) to the recorded I_d_ at each fixed V_g_ bias. For a certain combination of temperatures and gate biasing conditions, this analysis revealed a 1/*f*^*2*^ trend in the noise power spectral density (PSD) data. Specifically, the PSD at low temperatures exhibits a 1/*f*^2^ trend at the subthreshold regime (see Figure 2c green curve), a 1/*f* trend beyond V_th_ (see Figure 2c light blue curve) and 1/*f* trend for room temperature (see Figure 2c dark blue curve). The observation of the 1/*f*^*2*^ trend is the signature of a Lorentzian spectrum with two characteristic time constants, indicating the presence of a two-level RTS in the time domain^42−44^ (see Figure 3).

The observation of a 1/*f*^*2*^ noise in large (∼100 μm^2^) all-vdW FETs at cryogenic temperatures is intriguing, which can be explained phenomenologically with the aid of Figure 2d-f. In large-area FETs with low-disorder (i.e., low defect state density within the energy gap) gate dielectrics like hBN, alternate trapping and detrapping of electrons at a single energetically active defect species will induce two-level switching in current (I_d_) or RTS in the time domain. Regardless of its spatial quantity across the channel region, as schematically illustrated in Figure 2d, the RTS from this single defect species will manifest itself as a Lorentzian spectrum in the frequency domain.

In contrast, FETs with conventional disordered dielectrics, such as SiO_2_, exhibit multiple energetically active defect species with distinct characteristic time constants under a given LFN measurement condition. Figure 2e schematically illustrates such a device, where different colors denote a given energetically active defect species with distinct characteristic time constants. Each distinct defect species can result in a Lorentzian spectrum with a characteristic corner frequency that is nonoverlapping (see Figure 2f, spectra are color-coded the same as the defects symbols in Figure 2e). In such devices, the summation of multiple 1/*f*^*2*^ spectra, originating from defect states with distinct characteristic time constants and thus different corner frequencies, yields a PSD with 1/*f* trend (see red dashed line in Figure 2f).

With the above insights, the results in Figure 2c suggest that under certain LFN measurement conditions, only a single defect species in hBN is energetically active within several *k*_B_*T* of the Fermi level. Moreover, we attribute the transition from a 1/*f*^*2*^ to 1/*f* trend beyond the subthreshold regime to the increasing number of defect states from MoS_2_ within a few *k*_B_*T* of the Fermi level.

While the phenomenological model in Figure 2 offers a plausible explanation for our LFN observations, gaining a deeper understanding of the responsible defects requires further analysis. To guide our investigation into the nature of these defects, we pose two main questions: (1) Does the observed RTS originate from a single defect species across all bias and temperature conditions? (2) What is the physical location of these defects? We will answer these questions by performing statistical analyses of the RTS data in the next section. Subsequently, we will use the insight from our quantitative analyses into the microscopic properties of the defects to infer their identity through first-principle computational simulations.

### Energy and Location of Defects Inducing RTS in vdW MoS_2_ FET

As expected from the Lorentzian PSD in the frequency domain, our experiments displayed RTS or a two-level switching characteristic in the time domain, indicating the influence of only one defect energy level E_T_ on trapping and detrapping under each measurement condition (i.e., temperature and gate bias). To delve deeper into the nature of defects, we performed a quantitative analysis of the RTS data.

Initially, we extracted the variations of I_d_ amplitude (ΔI_d_) in time by evaluating the difference between low- and high-current levels. We then used these data sets to obtain the average time constants associated with the high and low current levels, i.e., τ_hi_ and τ_lo_, respectively. To do so, we followed a well-established process described in ref.^45^ Briefly, the two-level RTS can be treated as a discrete Markovian process with a Gaussian background. The high- and low-current levels can be redistributed into two Gaussian histograms centered at different signal amplitudes. The averaged time constant can be estimated from the area under the Gaussian curves divided by the number of transitions in between high- and low-current levels.^45^Supplementary Note 4 provides an example of the time constant extraction.

Recall that the objective of employing an n-type FET is to probe the trap energy levels closer to E_c_ by adjusting the gate bias. Therefore, we closely examined the effect of the gate bias on the dynamics of RTS (i.e., τ_hi_ and τ_lo_) at each measurement temperature. Figure 3a-d illustrate a few representative RTS data sets for measurements at the base temperature of 50 K at V_g_ = 2, 2.25, 2.5, 2.75 V. In this data set, the average time spent in the high-current state τ_hi_ reduced significantly as V_g_ increased, whereas the average time spent in the low-current state τ_lo_ followed an opposite trend. We associate the low-current state with the filled state since the probability of an electron occupying a defect state increases as V_g_ increases.

Temperature is another critical factor for energetically activating a defect state to generate RTS. Therefore, we additionally investigated the temperature dependence of RTS. Figure 3e-h show a few representative plots of the two-level RTS data sets, measured at different temperatures of 150, 166, 187, and 235 K and V_g_ = 2 V. These results qualitatively indicate the increase of the switching rate with temperature.

**Figure 4:**
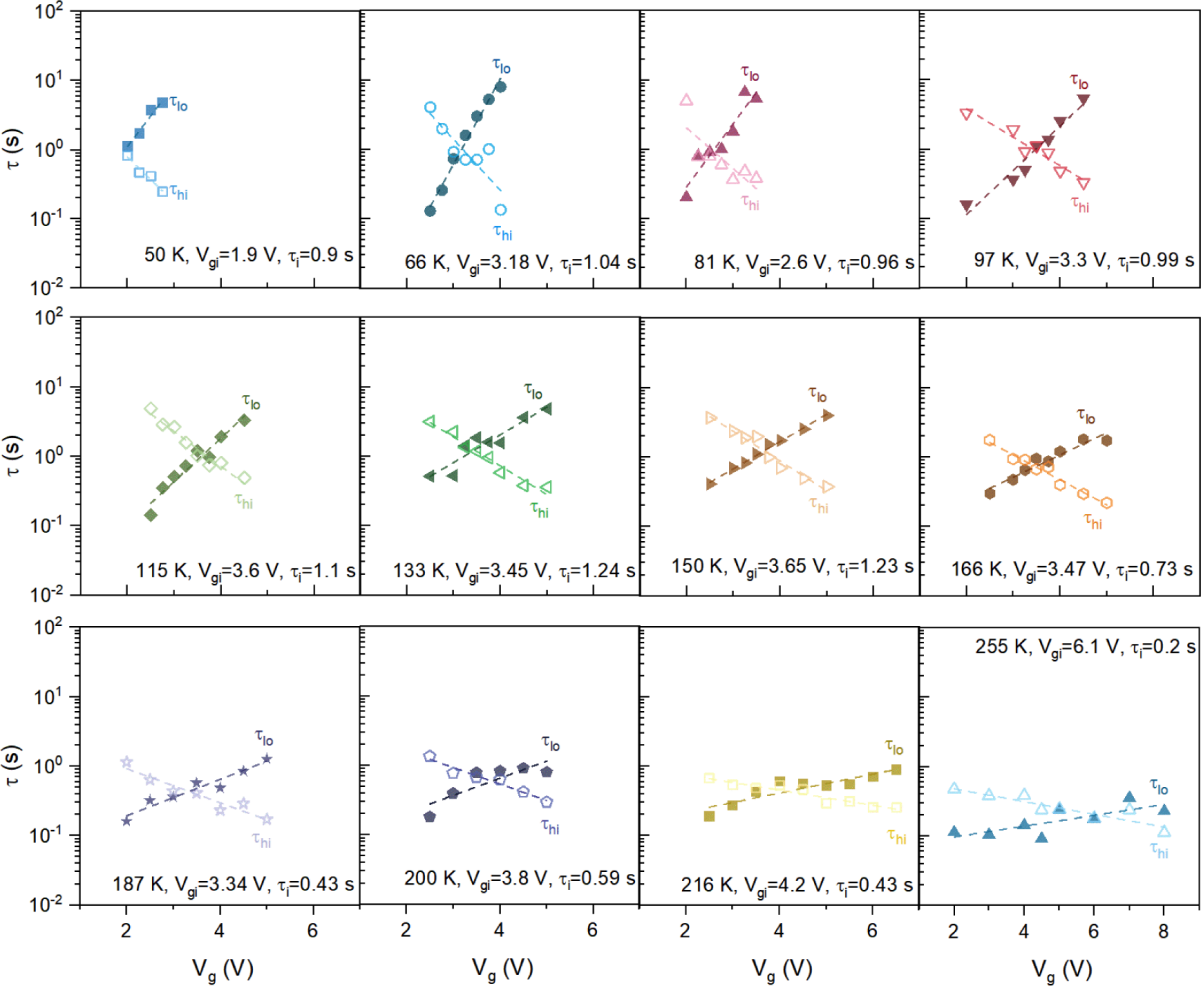
Time constants at different temperatures and V_g_ biases: Time constant extracted at different temperatures and gate biasing conditions from the observed RTS data. The averaged characteristic time constants were used for analyzing the energetic and spatial distributions of defects responsible for RTS.

**Figure 5:**
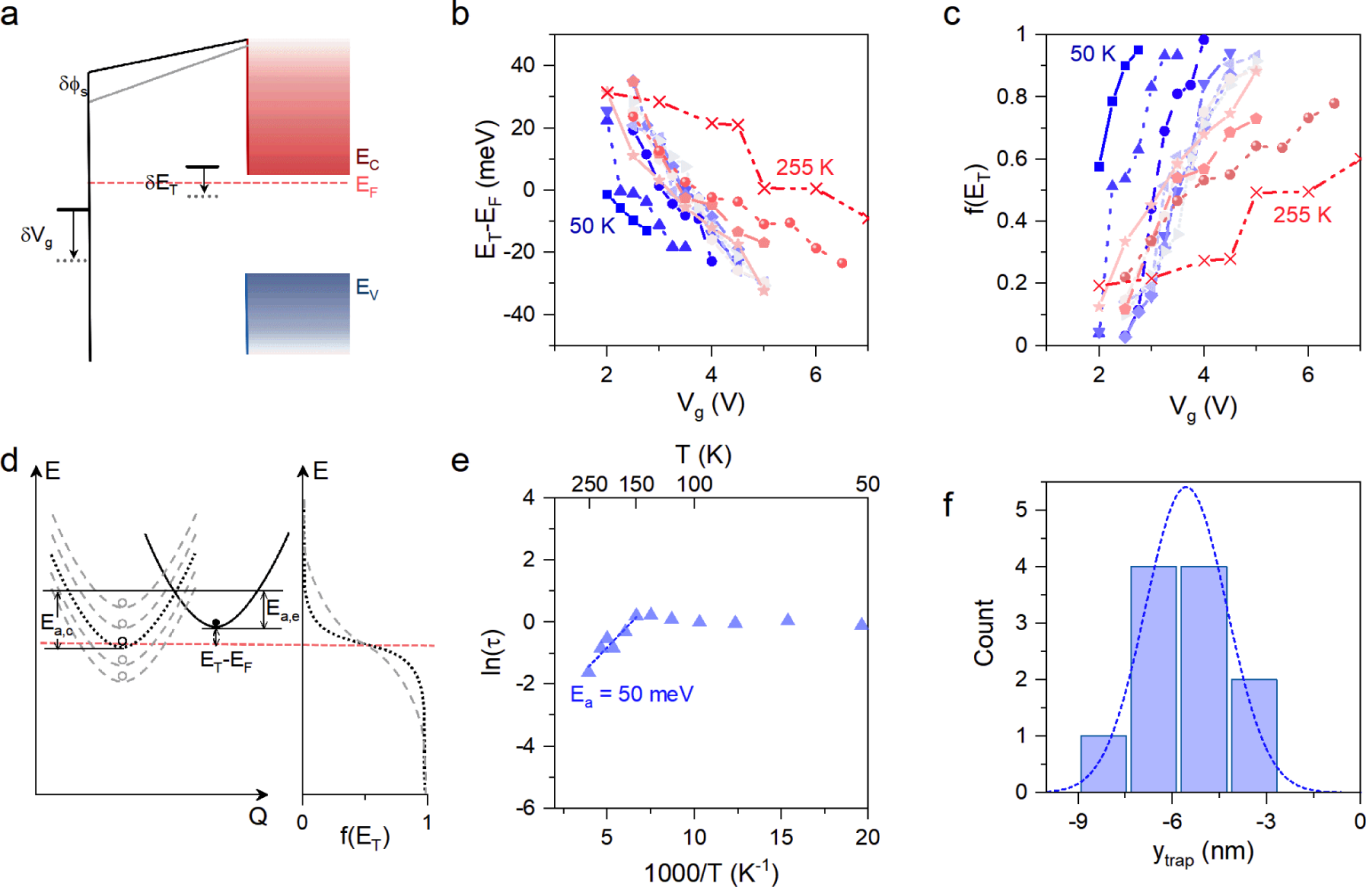
Energetic and spatial distributions of defects. (a) The band diagram illustrates the effect of V_g_ on shifting the trap energy level E_T_. (b) The calculated trap energy level relative to E_F_ and (c) corresponding trap occupancy as a function of V_g_ at different temperatures. (d) CCD schematically illustrates the defect dynamics at different temperatures. (e) The trap activation energy extracted by fitting the data at the high temperature regime (>150 K). (f) Distribution plot showing the spatial location of defects estimated from measurements at different temperatures. The negative sign indicates that defect sites are several nanometers inside hBN.

To examine the effect of bias and temperature on defect dynamics, we plotted the average time constants (τ_hi_ and τ_lo_) against the gate bias at different measurement temperatures. Figure 4 illustrates the summary of these plots. The observed V_g_ dependence of the time constants can be understood as schematically depicted in Figure 5a. As V_g_ becomes more positive, the defect energy level shifts downward. The energy separation between the defect state E_T_ and channel Fermi level E_F_ can be estimated at different V_g_ and temperatures using^23,43^
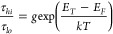
1where g is the trap degeneracy factor, and can be considered to be 1.^46,47^Figure 5b plots the corresponding trap energy level relative to the Fermi level (E_T_-E_F_) as a function of V_g_ obtained at different measurement temperatures. While this analysis tracks the changes in E_T_ relative to E_F_, the uncertainty in determining the position of E_F_ within the energy gap prevents the estimation of the absolute E_T_ level. Therefore, additional analysis is needed to examine whether these defect states causing the RTS originate from a single defect species.

To answer this question, we calculated the trap occupancy from the gate bias dependent E_T_-E_F_:^43^
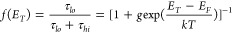
2

Figure 5c summarizes the extracted *f(E*_*T*_*)* against V_g_ at different temperatures. This analysis revealed the monotonic change of trap occupancy with V_g_. This characteristic of the trap occupancy provides strong evidence that the measured trap energy levels under different bias and temperature conditions originate from a single defect species.^48^

To fully understand the temperature-dependent trends of the data in Figure 5b-c, let us qualitatively consider the nonradiative multiphonon (NMP) process, illustrated schematically using the configuration-coordinate diagram (CCD) in Figure 5d. The broken curves (both dashed and dotted lines) with open circles represent available electron states around the Fermi level. The solid curve and full circle represent the final state of a filled trap after electron capture. *E*_a,c_ and *E*_a,e_ represent the activation energy of capture and emission, respectively. Therefore, the probability of capturing an electron depends on the distribution of the initial states, the activation energy, and the overlap with the final state. As depicted in the Fermi–Dirac plot, increasing temperature smears the distribution. Thus, one would expect the probability of capture to increase.

Since the gate bias modifies the trap energy level relative to the Fermi level, an equal energy barrier situation (i.e., *E*_a,c_=*E*_a,e_=*E*_a_) can be reached at a specific V_g_=V_gi_. Under such a condition, the probability of a defect site capturing or emitting an electron becomes equal, providing τ_hi_=τ_lo_=τ_i_. Figure 5e presents the plot of τ_i_ as a function of inverse T, which exhibits two distinct regimes: at *T* > 150 K, τ_i_ decreases with increasing T which is typical for a thermionic process; while at *T* < 150 K, τ_i_ remains nearly unchanged. The temperature independence of τ_i_ at *T* < 150 K is reminiscent of previous reports on conventional semiconductor FETs,^24^ and MoS_2_ on Al_2_O_3_ FET.^27,29^ Similar to those studies, we attribute this insensitivity of trap dynamics to the dominance of the tunneling mechanism at low temperatures.^49,50^ We will return to analyzing the quantum tunneling-dominated behavior later by computational MS-DFT methods. In the high-temperature regime (*T* > 150 K), however, the charge carrier capture and emission are thermally activated with an activation energy barrier *E*_a_. Therefore, we can extract the activation barrier *E*_a_ by fitting the data in the high-temperature regime using^51^
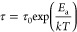
3where 1/τ_0_ is proportional to the capture cross section σ_0_. From our data, we extracted the thermally activated barrier as *E*_a_ = 50 meV.

Finally, we turn our attention to the second central question of our analysis, which seeks to determine the spatial location of the defect (y_trap_) that induces RTS. This information can be derived from studying how the charged trapping and detrapping affect the electrostatic field of the FET. Using a capacitance model and ignoring the surface potential,^48^ the distance between defect and channel (y_trap_) can be estimated as^43,52^
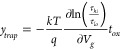
4where  is the equivalent dielectric thickness considering hBN and SiO_2_ in series. Applying this analysis to each data set in Figure 4, we estimated the defect location and plotted their distribution in Figure 5f. The data suggest that the trap levels responsible for RTS are located inside hBN and a few nanometers below its interface with MoS_2_.

### Atomistic Origins of RTS

The hBN lattice can accommodate a wide range of defect types,^18−21^ with the specific defects largely influenced by the growth conditions^21^ or the postgrowth processing, such as irradiation.^53^ In low-disorder single-crystal hBN, where external impurities have not been intentionally introduced, common defect types include native point defects, such as nitrogen vacancy (V_N_) and boron vacancy (V_B_), along with carbon-related impurity defects.^19−21^ Specifically, carbon impurity during hBN growth can be favorably formed by substituting for boron (C_B_) or nitrogen sites (C_N_) in the hBN lattice. Whereas V_B_ and C_N_ are acceptor type defects, C_B_ and V_N_ are donor type defects and energetically located in the upper half of the hBN bandgap.^21^ These characteristics of C_B_ and V_N_ align with our experimental findings, leading us to consider C_B_ and V_N_ as potential candidates in our computational analysis. Having selected these defect candidates, we performed MS-DFT calculations, which allows previously intractable first-principles gate bias simulations.^34,35,54,55^

**Figure 6:**
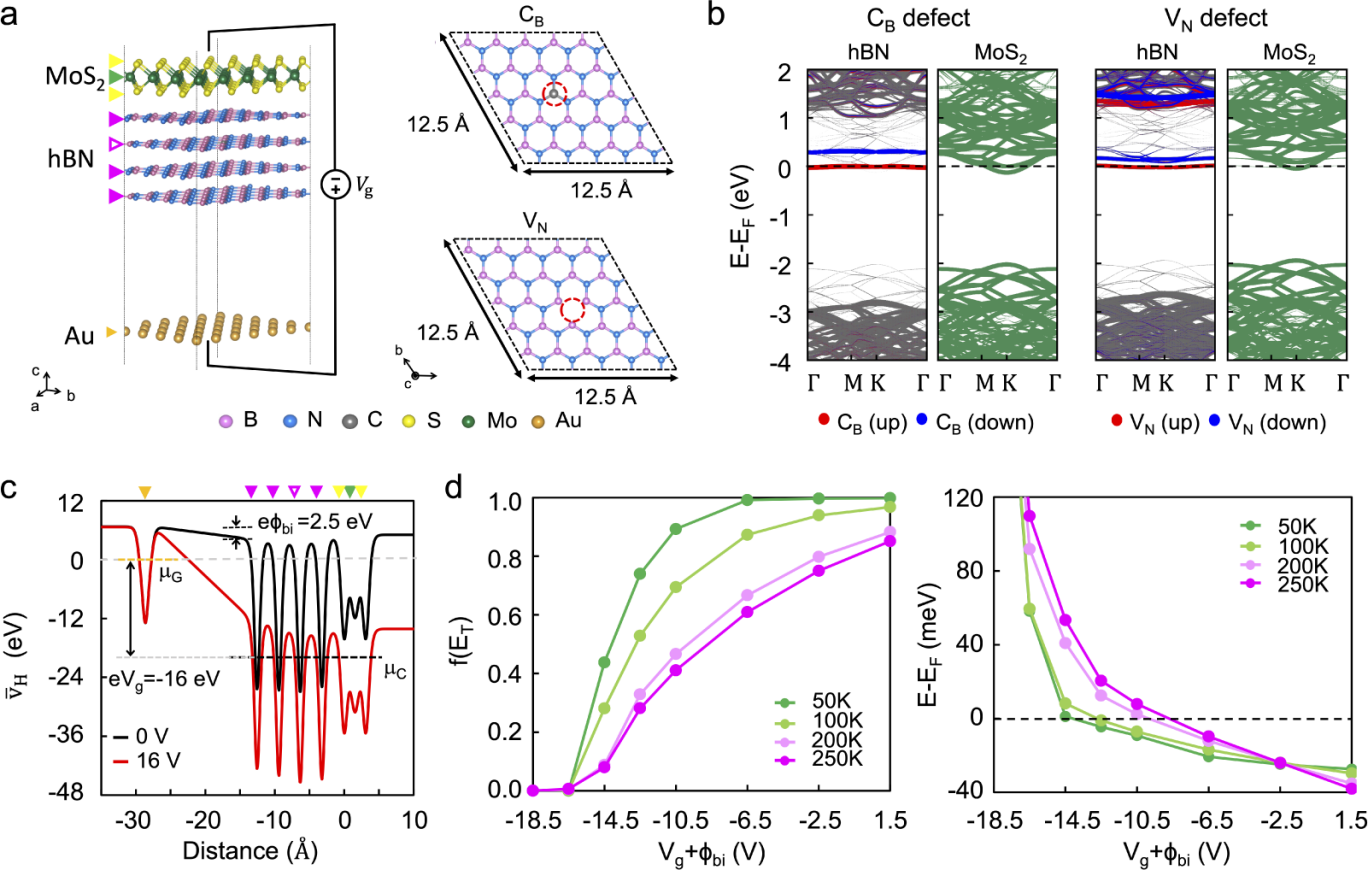
MS-DFT simulations of the gate response of defective hBN/MoS_2_ heterostructures. (a) The calculation model of Au/hBN/MoS_2_-based 2D FET structure (left panel). C_B_ or V_N_ defect (right panel) was introduced into the second interfacial hBN layer. (b) The equilibrium (V_g_ = 0 V) band structures projected onto MoS_2_ and hBN with C_B_ (left) and V_N_ defects (right). The red and blue circles represent the two spin-polarized states of C_B_ and V_N_ defects, respectively. The size of the circles quantifies the strength of the orbital contribution. (c) The plane-averaged electrostatic potential energies calculated for V_g_ = 0 V (black line) and −16 V (red line) at *T* = 250 K. (d) The occupations (left panel) and energetic positions (right panel) of C_B_ as a function of V_g_ obtained at *T* = 50, 100, 200, and 250 K.

In Figure 6a, we present the atomic model of a capacitor based on the hBN/MoS_2_ heterostructure, featuring a monolayer Au gate electrode, a four-layer hBN dielectric, and a monolayer MoS_2_ FET channel (see Methods and Supplementary Note 5 for details). The V_N_ and C_B_ defects were placed in the second hBN layer from the MoS_2_, as denoted by an open triangle in Figure 6a,c. The equilibrium band structures shown in Figure 6b reveals that in both cases *E*_*F*_ is located at the lower spin-polarized donor-type C_B_ or V_N_ defect states, which are pinned to the MoS_2_ conduction band minimum (marked with dashed line in Figure 6b). In Figure 6c, we present the plane-averaged Hartree electrostatic potential  obtained from an equilibrium DFT calculation and an MS-DFT calculation at V_g_=-16 V. The usage of Au monolayer as the gate electrode resulted in a built-in potential of ϕ_bi_=-2.5 V in equilibrium, or the flat-band condition corresponds to V_g_=+2.5 V. We summarize in Figure 6d the calculated trap occupations *f(E*_*T*_*)* and the energetic position E_T_ relative to E_F_ of the C_B_ defect obtained at different gate bias and temperature conditions. With the V_N_ defect, we obtained similar *f(E*_*T*_*)* and E_T_-E_F_ data. Because the periodic boundary conditions imposed along the hBN plane directions result in a defect density much larger than that in the experimental situation, the applied V_g_ values in our calculations are much larger than those in the experiment. Other than this quantitative difference, however, the good qualitative comparison with experimental data (Figure 5b,c) indicate that our first-principles calculations indeed properly capture the mechanistic origins of the RTS observed in experiments and justify C_B_ and V_N_ as the defect candidates that produce the experimentally observed RTS.

**Figure 7:**
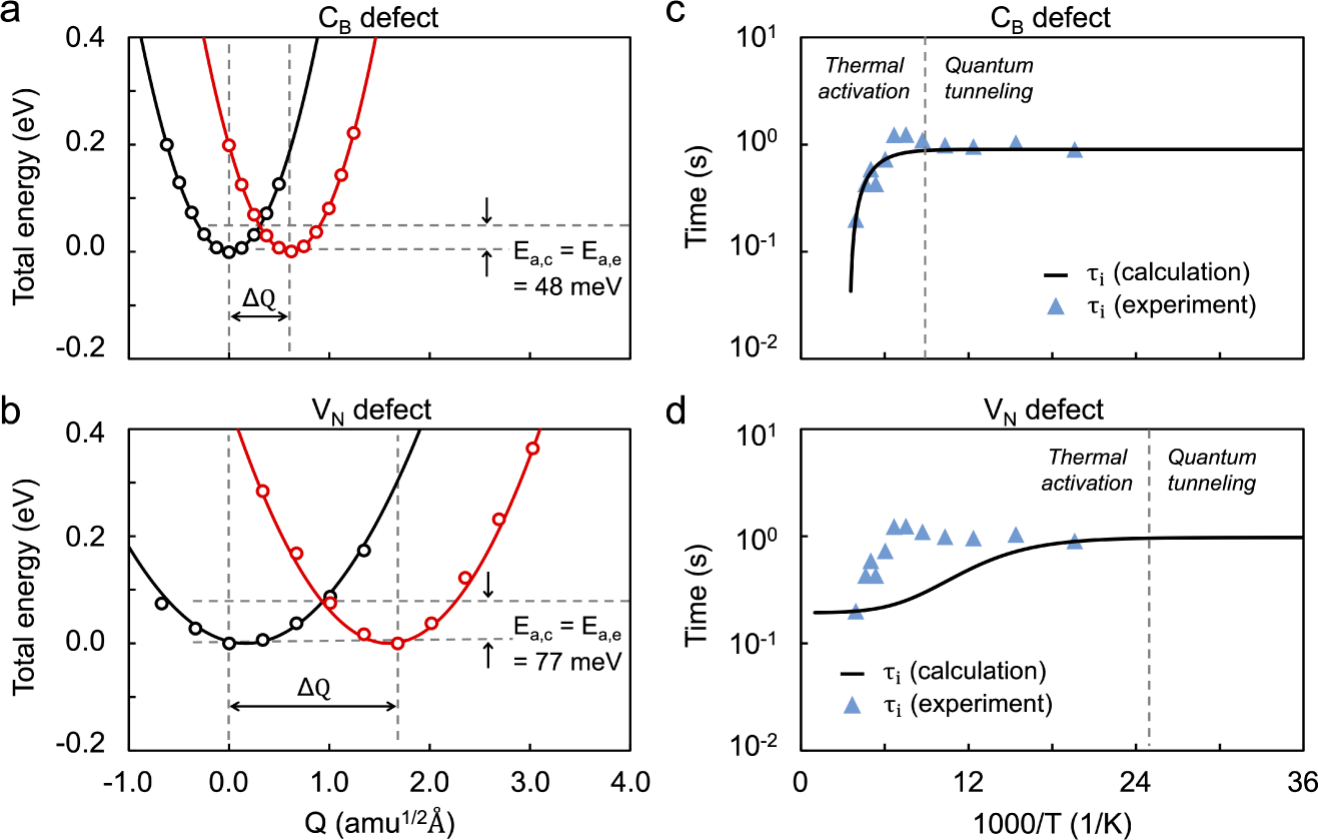
Identification of the C_B_ defect as the atomistic origins of RTS. The CCDs of the (a) C_B_ and (b) V_N_ defects calculated at *T* = 250 K within the zero-charge (black curves) and +1*e* charge (red curves) constraints. Unfilled circles represent the data points explicitly obtained from MS-DFT calculations and solid lines are the second-order polynomial fits. The calculated (black line) time constant τ_i_ values obtained by fitting the NMP model with parameters for the (c) C_B_ and (d) V_N_ defects to the *T* = 50 and 255 K experimental τ_i_ values. Vertical gray dotted lines indicate the transition points between temperature-dependent to temperature-independent regimes, which correspond 3% deviation points from *T* = 50 K τ_i_ values.

To pinpoint the atomistic origin of the RTS between C_B_ and V_N_, we next analyzed the hBN trilayer model gated by a Au monolayer and examined the structural deformations associated with nonradiative charge trapping and detrapping in C_B_ and V_N_ defects (see Methods). Performing constant-charge 0*e* and +1*e* MS-DFT calculations, which roughly correspond to constant-gate bias V_g_=+2.5 V and V_g_=-16 V MS-DFT calculations, respectively, we first optimized the respective geometries and then calculated the potential energy surfaces as a function of the mass weighted distortion or configuration coordinate (*Q*). In Figure 7a,b, we summarized the calculated CCDs for C_B_ (top) and V_N_ (bottom) at the crossover voltage V_g_=V_gi_. We then performed NMP model analyses as follows.^56−58^ First, we calculated the distance in the configuration coordinate
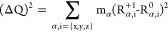
5where *m*_*α*_ is the atomic mass of atoms α and  is the atomic coordinates of the optimized geometries in the neutral (0) and charged (+1) states, respectively (see Methods). Defining the modal mass of the vibration *M = (*Δ*Q)*^*2*^*/*(Δ*R)*^*2*^, where , we next extracted the effective phonon energies (ℏΩ_0/+1_) and Huang–Rhys factors (S_0/+1_) from the 1D CCDs of the neutral and charged states (Table 1). The calculated Δ*Q* are 0.62 and 1.68 amu^1/2^ Å, with the corresponding Huang–Rhys factor *S*_*c*_ of 2.94 and 9.54 for C_B_ and V_N_, respectively. This analysis indicates a much stronger electron–phonon coupling in V_N_ than C_B_.

**Table 1:** NMP Model Parameters for the C_B_ and V_N_ Defect Cases

Defect	Δ*Q* (amu^1/2^ Å)	*M* (amu)	ℏΩ_0_ (meV)	ℏΩ_+1_ (meV)	*S*_0_	*S*_*+1*_
C_B_	0.62	11.80	63.84	65.46	2.94	3.02
V_N_	1.68	11.98	28.21	31.55	9.54	10.67

For more direct comparisons between computational and experimental data, we then calculated the crossover lifetimes *τ*_*i*_ at V_g_=V_gi_ for C_B_ and V_N_ cases as functions of temperature by fitting to the experimental *τ*_*i*_ values at *T* = 255 and 50 K. The results summarized in Figure 7c,d for the C_B_ and V_N_ cases both show the transitions between temperature-dependent and temperature-independent *τ*_*i*_ behaviors at high-temperature and low-temperature regimes, respectively. As discussed above, the former and latter can be attributed to thermal activation and nuclear quantum tunneling regimes, respectively. Moreover, we found that the transition temperature of the C_B_ case (130 K) is much closer to the experimental value (150 K) than that of the V_N_ counterpart (40 K). Additionally, we obtained the *E*_a_ values of 48 and 77 meV for the C_B_ and V_N_ cases, respectively, and the C_B_ case value again provides a much better agreement with the experimentally estimated value of 50 meV. Furthermore, as mentioned earlier, the formation of C_B_ is thermodynamically more favorable than V_N_.^20^ Therefore, we conclude that among the two defect candidates C_B_ is the atomistic origin of the experimentally observed RTS.

## Conclusions

The results established here demonstrate the utility of a hBN-encapsulated and graphene-contacted low disorder MoS_2_ FET for probing the defect dynamics in hBN. Despite the large area (100 μm^2^) of our FET, our LFN measurements revealed discrete-level switching in the transistor current at cryogenic temperatures, indicating charging and discharging of the defect levels associated with a single defect species within hBN. The experimental data revealed the positively charged nature of the defect and its energetic and spatial distribution. Combining the LFN spectroscopy experimental data with theoretical MS-DFT analysis, we assigned the carbon atom substituted into the boron site in single-crystal hBN as the most likely defect candidate.

As future research directions, a similar device structure can be created using a p-type channel material to probe the acceptor type defects that is close to the valence band edge of hBN. Moreover, capacitance measurement using sufficiently large hBN flake would allow defect characterization throughout its band gap, similar to past studies performed on MoS_2_.^59,60^ Furthermore, irradiating hBN to create known defect types could be helpful in revealing their LFN behavior and ascertain the atomic origin of RTS. Advanced understanding of hBN defects achieved in this work will be beneficial for further improvements in material quality.^61^ We also anticipate that these findings can inform research on defect engineering for future electronic and quantum device applications.

## Methods

### Device Fabrication and Characterization

Standard nanofabrication was used for fabrication of devices from the hBN-encapsulated MoS_2_ heterostructures. The details of the 2D heterostructure construction and device fabrication are given in Supporting Information. All electrical measurements were performed in dark under vacuum conditions in a Lakeshore probe station. The transfer and output characteristics were obtained using Keysight B1500 parameter analyzer under the normal mode with a step size of 20 mV. In the I_d_-V_g_ curves, we observed 20 mV of hysteresis at I_d_ = 1 nA, which is equivalent to a trap charge density on the order of 10^9^ cm^–2^. Therefore, we concluded that the hysteresis is negligible. The transport properties were characterized using standard lock-in measurement. The LFN data were measured by a low-noise current preamplifier SR570. The devices were initialized prior to LFN measurements by applying a sufficiently low negative bias below the threshold voltage (i.e., V_gs_ < V_th_) to the gate electrode for a few minutes, while the other device terminals were connected to the ground (i.e., V_s_=V_d_ = 0 V).

### DFT and MS-DFT Calculations

To calculate the *f*_*T*_ and E_T_-E_F_ as functions of V_g_ and T, we modeled the hBN/MoS_2_-based capacitor by the single-layer Au/16 Å vacuum/four-layer hBN/single-layer MoS_2_ heterostructure and introduced the C_B_ or V_N_ defect into the second interfacial hBN layer. To minimize the lattice mismatch between hBN and MoS_2_, we scaled the 5 × 5 supercell of hBN (12.51 Å) and the 5 × 5 supercell of Au(111) (13.00 Å) to fit the 4 × 4 supercell of MoS_2_ (12.50 Å). A 2 × 2 Monkhorst–Pack k-point grid was sampled. For the CCD analysis, we utilized the Au/16 Å vacuum/3-layer hBN heterostructure model and introduced the C_B_ or V_N_ defect into the central hBN layer.

We performed spin-polarized DFT calculations using the SIESTA package.^62^ We employed the local density approximation (LDA),^63^ Troullier-Martins-type norm-conserving pseudopotentials,^64^ numerical atomic orbital basis sets of double-ζ-plus-polarization quality with a confinement energy of 20 meV, and a real-space integration mesh with the grid cutoff of 400 Ry. Atomic geometries were optimized until the ionic forces on each atom are reduced below 0.04 eV/Å. To describe radiative multiphonon emission processes, it would be necessary to employ more advanced exchange-correlation functionals such as hybrid functionals and correct LDA bandgaps and single-electron spectra.^56,58^ However, we expect LDA is sufficient for our purpose of obtaining the CCDs for NMP model analyses presented below.^57,58^

### Nonradiative Multiphonon Model Analyses

To evaluate *τ*_*i*_ for a defect based on the NMP model, we first approximated the MS-DFT-derived CCDs or potential energy surfaces of capture (c) and emission states (e) by 1D parabolic curves. Then, we evaluated the effective frequency of the capture/emission state according to
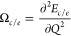
6and the corresponding Huang–Rhys factor, which quantifies the strength of electron–phonon coupling, according to
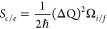
7

Using the ΔQ, *M*, and ℏΩ_c/e_

parameters (Table 1), we evaluated the transition rate *k*_*ce*_ between capture and emission states according to
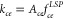
8which consists of the electronic part
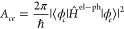
9where  is the perturbing Hamiltonian and *Φ*_*c/e*_ is the capture/emission-state electronic wave function, and the vibronic part (line shape function)
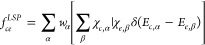
10where *w*_*α*_ is the thermal occupation of the vibrational state α, *X*_*c,α(e,β)*_ is the ionic wave function of the capture vibrational state α (emission vibrational state β), and E_*c,α(e,β)*_ is the energy level of the capture vibrational state α (emission vibrational state β), which were analytically determined for the two harmonic defect states. Based on the NMP parameters derived from the C_B_ and V_N_, we finally fitted 1/ to the *T* = 50 and 255 K experimental τ_i_ values.

## Data Availability

Data supporting the findings of this manuscript are available from the corresponding author upon reasonable request.

## Supporting Information Available

Additional experimental details, including material production, materials characterization, and device analysis are provided (PDF)
