# Supplementary material for: Characterizing Defects Inside Hexagonal Boron Nitride Using Random Telegraph Signals in van der Waals 2D Transistors
Source: ACS Nano. 2024 Sep 28;18(42):28700–11. doi: 10.1021/acsnano.4c06929 (PMC11503768; doi:10.1021/acsnano.4c06929)
Supplement: Supplementary file 1 — nn4c06929_si_001.pdf [file nn4c06929_si_001.pdf]

Supplementary Materials for

Characterizing defects inside hexagonal boron nitride using random telegraph signals in van der Waals 2D transistors

Zhujun Huang[1], Ryong-Gyu Lee[2], Edoardo Cuniberto[1], Jiyoon Song[2], Jeongwon Lee[2], Abdullah Alharbi[1,3], Kim Kisslinger[4], Takashi Taniguchi[5], Kenji Watanabe[6], Yong-Hoon Kim*,[2], Davood Shahrjerdi*,[1]

1 Electrical and Computer Engineering, New York University, Brooklyn, NY 11201
2 School of Electrical Engineering, Korea Advanced Institute of Science and Technology (KAIST), 291 Daehak-ro, Yuseong-gu, Daejeon 34141, Korea
3 Microelectronics and Semiconductor Institute, King Abdulaziz City for Science and Technology (KACST), Riyadh 11442, Saudi Arabia
4 Center for Functional Nanomaterials, Brookhaven National Laboratory, Upton, NY 11973, USA
5 Research Center for Materials Nanoarchitectonics, National Institute for Materials Science, 1-1 Namiki, Tsukuba 305-0044, Japan
6 Research Center for Electronic and Optical Materials, National Institute for Materials Science, 1-1 Namiki, Tsukuba 305-0044, Japan

* Corresponding authors: y.h.kim@kaist.ac.kr, davood@nyu.edu

Supplementary Note 1. Fabrication of all vdW MoS_2 field-effect transistors. We prepared the hBN and graphene flakes by direct exfoliation onto a SiO_2/Si substrate. Whereas few-layer MoS_2 flakes were obtained by exfoliating onto a poly(vinyl) alcohol (PVA) film-coated substrate to improve the exfoliation yield and achieve large-size flakes (1). The transfer process started by picking up the top hBN flake using an elastomer. Then we used the top hBN flake to pick up graphene flakes with the desired alignment for the designer structures (see Figs. S1a, b). Subsequently, the MoS_2 flake was released from the substrate onto the hBN/graphene stack by locally dissolving the PVA coating underneath (see Fig. S1c). The complete hBN/graphene/MoS_2/hBN heterostructure was finished by laminating the top hBN/graphene/MoS_2 stack onto the bottom hBN flake at elevated substrate temperatures (2) (see Fig. S1d).

For this study, we used MoS_2 flakes with thicknesses ranging from 3-6 layers. We chose graphene electrical contact for two reasons: one is to form low ohmic contact between graphene and MoS_2 (3); the second is to preserve the MoS_2 channel material properties by encapsulation and avoid inducing defects during the deposition of metal contact (4, 5).

The alignment of graphene flakes ensures partial overlap with the MoS_2 flake, making electrical contact between graphene and MoS_2 but leaving channel area to be hBN-encapsulated MoS_2 only. Fig. S1 shows the schematics of building a two-terminal device structure. Fig. S2a shows an optical image of a four-point device. Note that we did not construct Hall-bar structure knowing that interface contaminants are likely to be trapped at the vdW flake edges. Therefore, we intentionally left one edge of the MoS_2 flake without graphene contact to achieve clean interfaces by high-temperature lamination.

Supplementary Note 2. Material characterization of hBN-encapsulated MoS_2. We further analyze the material properties of the all-vdW MoS_2 FET. The optical image in Fig. S2a shows no visible blisters formation in the active device area. The white dashed lines highlight the position of graphene flakes that overlap with few-layer MoS_2 before the RIE etch and form electrical contact to the metal leads. The black dashed lines highlight the device active region.

To characterize the interface quality, we first used Raman spectroscopy to probe the heterostructure homogeneity. In Fig. S2b, we show a representative Raman spectrum of few-layer MoS_2. Figs. S2c-d plots the spatial map of the peak positions of in-plane vibration (E_{2g}) and out-of-plane lattice expansion (A_{1g}) modes in the device active region, respectively. The narrow distribution of the two modes indicates homogeneous material properties.

Finally, in Fig. S3-4, we show the corresponding elemental components by electron dispersive X-ray spectroscopy (EDS) to the HRTEM images plotted in Figures. 1b-c in the main text. The EDS maps confirm the composition of the heterostructure and their positions.

Supplementary Note 3. Interface trap density extraction. We extracted interface trap capacitance (C_{it}= q^2D_{it}) from the SS equation, which assumes a uniform interface trap density distribution D_{it} within the gap. Specifically, the SS equation is given by (6)

$$SS = 2.3 \times \frac{k_B T}{q}\left(1 + \frac{q^2 D_{it}}{C_{ox}}\right)$$

where k_B is the Boltzmann constant, T is temperature (300 K), and q is the elementary charge. To extract the interface trap density for the device reported in Figure 1, we used the gate dielectric capacitance of C_{ox}=10^{-8} F.cm^{-2}. The gate dielectric consists of 285 nm SiO_2 and 30 nm hBN. We used dielectric constant of $\varepsilon(SiO_2)$=3.9 and ε(hBN)=3.

Supplementary Note 4. Estimation of defect trapping and de-trapping time constant. To extract statistically averaged time constant, it is important to acknowledge the limit in the noise spectroscopy setup. In our measurement, a sampling rate of 10,000 is always implemented. Therefore, we are not able to resolve time constant that is a few or sub-milliseconds reliably. Moreover, to ensure a reasonable estimation of the time constant, we would use the time domain RTS data that stores 50-100 level transitions. For example, extracting τ~1 s would require the recorded RTS data to be a few minutes; whereas τ~0.1 s can be obtained from the data set that is tens of seconds. In our measurement, the total recorded time domain data was always 3 minutes. Therefore, an estimation of τ ~10 s is likely to have more statistical error.

We implemented a previously developed algorithm for determining the statistical values of the averaged time constant (7). In Fig. S5, we show an example.

Supplementary Note 5: The location of the C_B defect in hBN layer near MoS_2. To illustrate the effect of spatial distance between the C_B defect in the hBN layer and the MoS_2 channel, we consider the three Au/4-layer hBN/MoS_2 models, which contain the C_B located at the (i) interfacial, (ii) second, or (iii) third hBN layer, respectively (Fig. S6). We present the orbital-projected band structures of these models under equilibrium conditions at T = 250 K. For (i), the C_B states are dispersively located near the CBM of MoS_2, and their wavefunctions strongly hybridize to MoS_2 channel. On the contrary, defect at the second (ii) and third (iii) layer show rather located C_B states whose wavefunctions are significantly separated from the MoS_2. Accordingly, only the localized C_B states in the (ii) or (iii) can be characterized as broader trap potentially contributing to the RTS, whereas the delocalized states in (i) are served as interfacial trap due to its fast carrier relaxation time. For the computational analysis of RTS, we selected the (ii) model in the manuscript as the representative case.

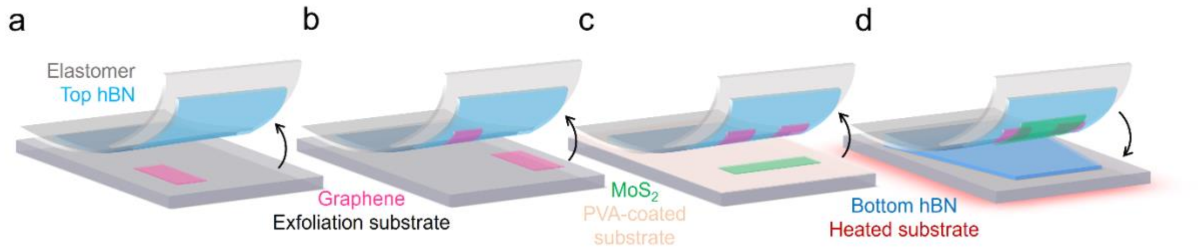

Fig. S1. Stacking steps of the all-vdW heterostructures: (a) Top hBN flake was first picked up by an elastomer. Then the elastomer/hBN stack was used to pick up the first graphene flake as one of the electrical contacts to MoS_2. (b) Subsequent pick-up of the graphene flakes with desired alignment. In this case, we show an example of a two-terminal structure. (c) The elastomer/hBN/graphene stack was used to pick up PVA-exfoliated MoS_2 flake. This step is similar to ref (2). (d) Final high-temperature lamination step to remove interfacial contaminants and complete the all-vdW heterostructure.

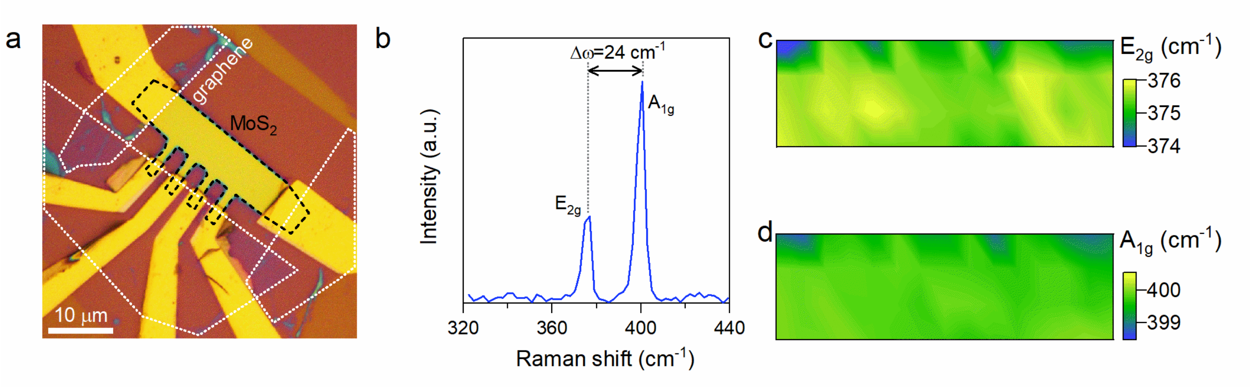

Fig. S2. Material characterization of the all-vdW heterostructure: (a) Optical image of a four-point structure all-vdW MoS_2 FET. (b) A representative Raman spectrum of few-layer MoS_2 in the hBN-encapsulated channel region. (c)-(d) Spatial Raman map of peak positions of the in-plane (E_{2g}) and out-of-plane (A_{1g}) modes suggest homogeneous interfaces.

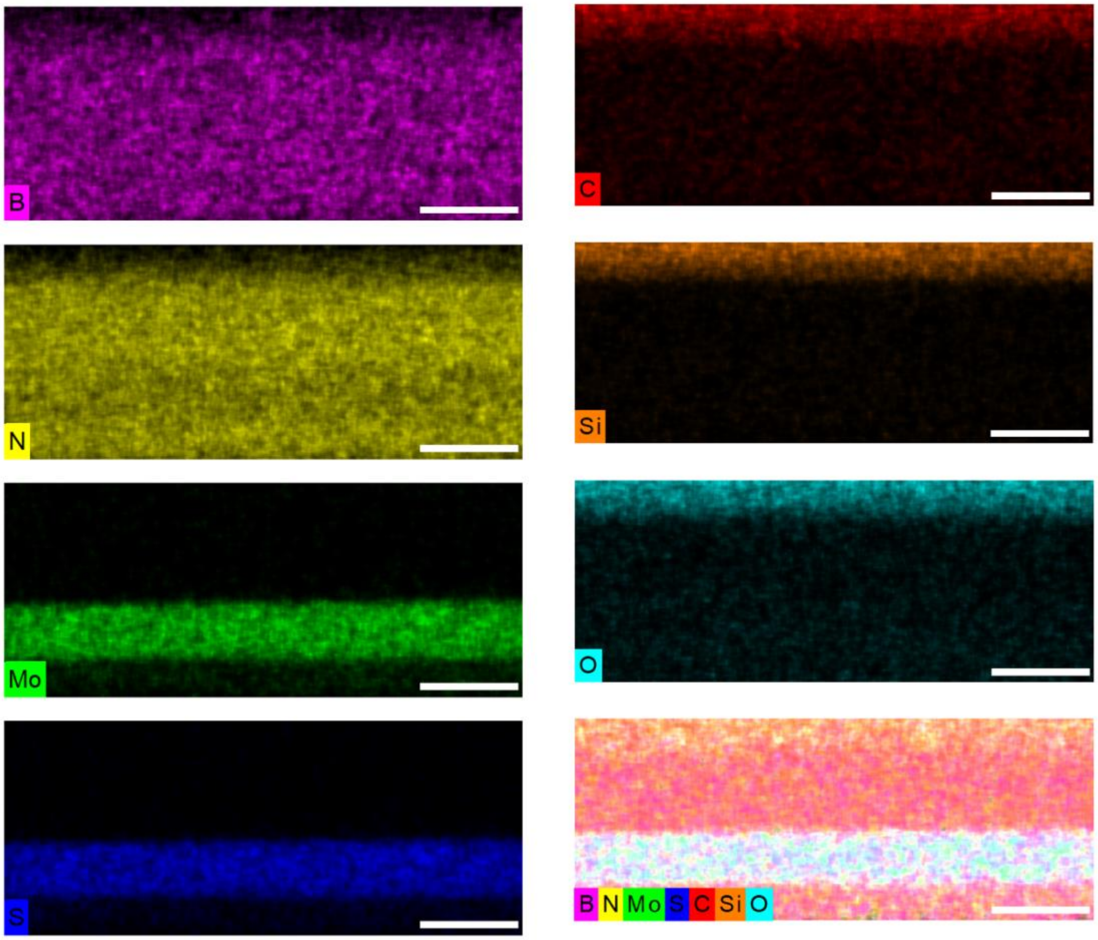

Fig. S3. EDS maps at the channel region. The scale bar is 20 nm.

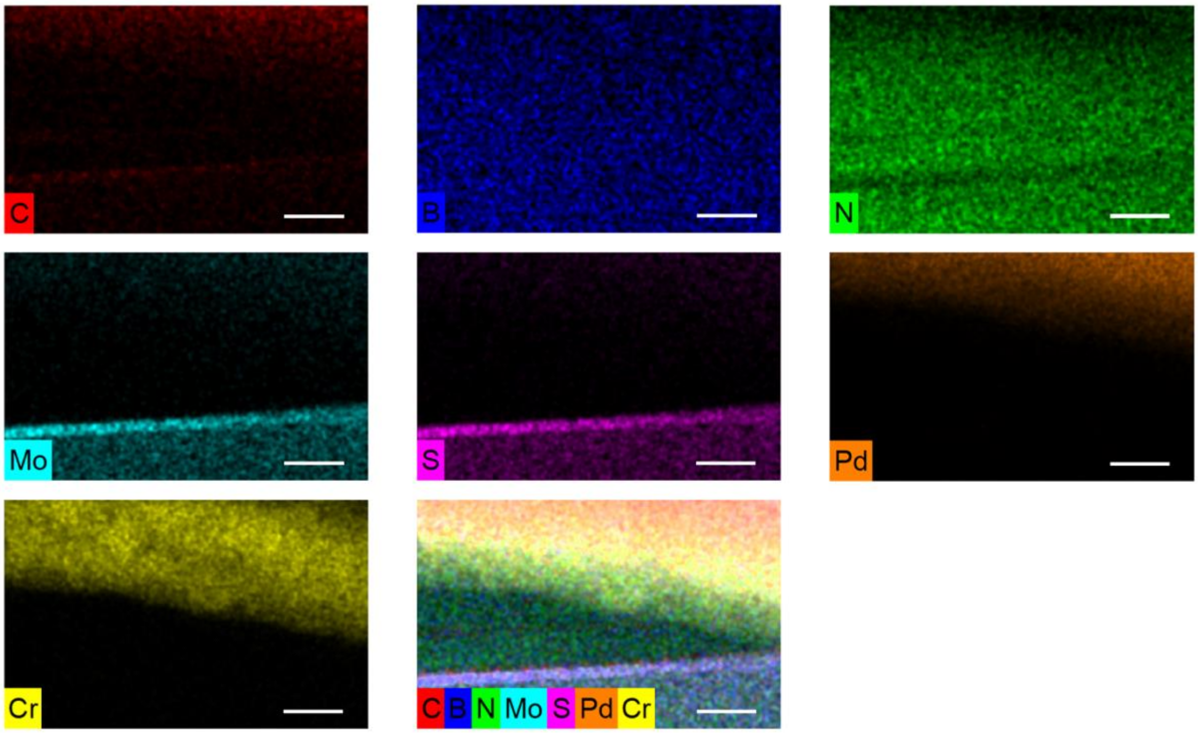

Fig. S4. EDS maps at the contact region. The scale bar is 10 nm.

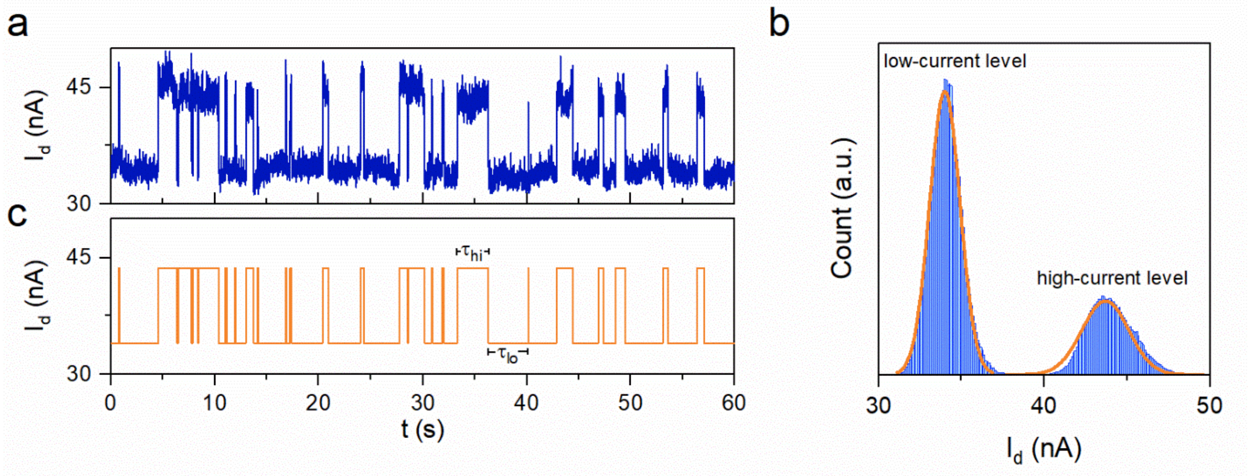

Fig. S5. Time constants extraction: (a) Example of the drain current measured during the noise spectroscopy shows discrete two-level switching behavior. The measurement was taken at 150 K, V_g=4 V and V_d=1 V. (b) The amplitude histogram plot (blue bars) of I_d in panel (a) shows well-separated two sub-records that exhibit Gaussian distribution indicating a two-level system. The orange curves show the fitted Gaussian distributions. (c) The recovered drain current shows good agreement with experimental data in panel (a). Following ref (7), we calculated the individual switches and durations and extracted the average time constant of high- (τ_{hi}=0.66 s) and low-current levels (τ_{lo}=1.65 s).

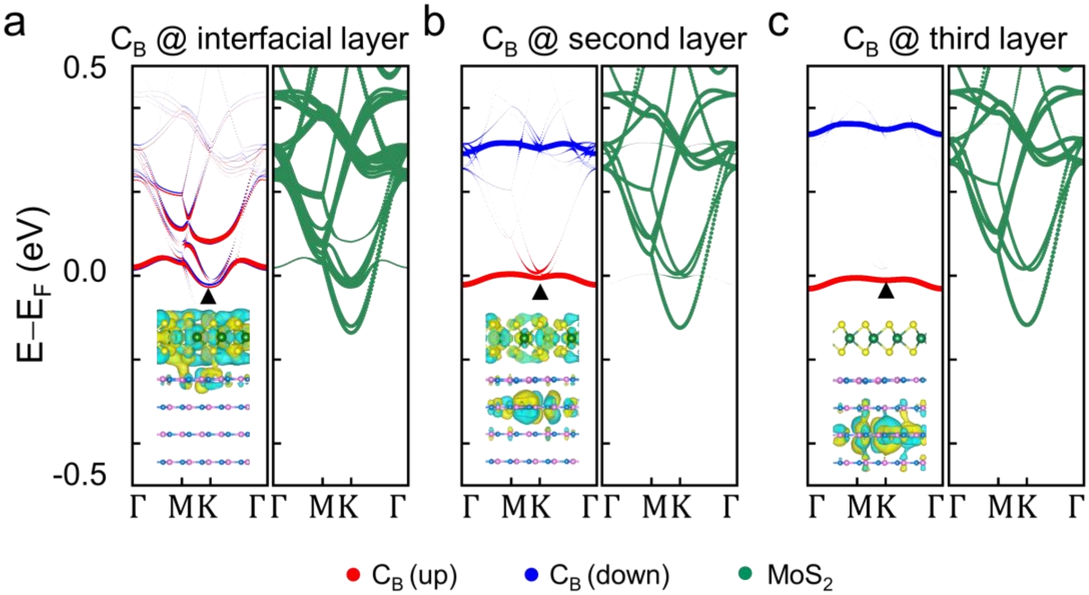

Fig. S6. The location of the C_B defect in hBN layer near MoS_2 channel: The projected band structure and the wavefunctions of the C_B defect are obtained. The defect is introduced into (a) the interfacial layer, (b) the second layer and (c) the third layer in hBN near the MoS_2 channel. In the figure, the green, red and blue circles represent the MoS_2, the up-spin state, and the down-spin state of the C_B defect, respectively. The size of the circles quantifies the strength of the orbital contributions. The black triangle indicates the position of the C_B defect at K-point of k-path for the band structure. The wavefunctions project the position of black triangle, and the iso-surface level is adopted as 0.02 $Å^{-3}$.

References

1. Huang Z, Alharbi A, Mayer W, Cuniberto E, Taniguchi T, Watanabe K, et al. Versatile construction of van der Waals heterostructures using a dual-function polymeric film. Nature communications. 2020;11(1):3029.
2. Huang Z, Cuniberto E, Park S, Kisslinger K, Wu Q, Taniguchi T, et al. Mechanisms of Interface Cleaning in Heterostructures Made from Polymer-Contaminated Graphene. Small. 2022;18(20):2201248.
3. Cui X, Lee G-H, Kim YD, Arefe G, Huang PY, Lee C-H, et al. Multi-terminal transport measurements of MoS2 using a van der Waals heterostructure device platform. Nature nanotechnology. 2015;10(6):534-40.
4. Liu L, Kong L, Li Q, He C, Ren L, Tao Q, et al. Transferred van der Waals metal electrodes for sub-1-nm MoS2 vertical transistors. Nature Electronics. 2021;4(5):342-7.
5. Liu Y, Guo J, Zhu E, Liao L, Lee S-J, Ding M, et al. Approaching the Schottky–Mott limit in van der Waals metal–semiconductor junctions. Nature. 2018;557(7707):696-700.
6. Sze SM, Li Y, Ng KK. Physics of semiconductor devices: John wiley & sons; 2021.
7. Yuzhelevski Y, Yuzhelevski M, Jung G. Random telegraph noise analysis in time domain. Review of Scientific Instruments. 2000;71(4):1681-8.
